# A novel esterase gene cloned from a metagenomic library from neritic sediments of the South China Sea

**DOI:** 10.1186/1475-2859-10-95

**Published:** 2011-11-09

**Authors:** Qing Peng, Xue Zhang, Meng Shang, Xu Wang, Guili Wang, Bingxue Li, Guohua Guan, Ying Li, Youshao Wang

**Affiliations:** 1 State Key Laboratories for Agro-biotechnology and College of Biological Sciences, China Agricultural University, Beijing, 100193, P. R. China; 2 State Key Laboratory of Tropical Oceanography, South China Sea Institute of Oceanology, Chinese Academy of Sciences, Guangzhou, 510301, P. R. China

**Keywords:** metagenomic library, functional screening, esterase, South China Sea

## Abstract

**Background:**

Marine microbes are a large and diverse group, which are exposed to a wide variety of pressure, temperature, salinity, nutrient availability and other environmental conditions. They provide a huge potential source of novel enzymes with unique properties that may be useful in industry and biotechnology. To explore the lipolytic genetic resources in the South China Sea, 23 sediment samples were collected in the depth < 100 m marine areas.

**Results:**

A metagenomic library of South China Sea sediments assemblage in plasmid vector containing about 194 Mb of community DNA was prepared. Screening of a part of the unamplified library resulted in isolation of 15 unique lipolytic clones with the ability to hydrolyze tributyrin. A positive recombinant clone (pNLE1), containing a novel esterase (Est_p1), was successfully expressed in *E. coli *and purified. In a series of assays, Est_p1 displayed maximal activity at pH 8.57, 40°C, with ρ-Nitrophenyl butyrate (C_4_) as substrate. Compared to other metagenomic esterases, Est_p1 played a notable role in specificity for substrate C_4 _(*k*_cat_/*K*_m _value 11,500 S^-1^m M^-1^) and showed no inhibited by phenylmethylsulfonyl fluoride, suggested that the substrate binding pocket was suitable for substrate C_4 _and the serine active-site residue was buried at the bottom of substrate binding pocket which sheltered by a lid structure.

**Conclusions:**

Esterase, which specificity towards short chain fatty acids, especially butanoic acid, is commercially available as potent flavoring tools. According the outstanding activity and specificity for substrate C_4_, Est_p1 has potential application in flavor industries requiring hydrolysis of short chain esters.

## Background

Marine microbes are a large and diverse group, and are exposed to a wide variety of pressure, temperature, salinity, nutrient availability, and other environmental conditions [1-3]. They provide a huge potential source of novel enzymes with unique properties that may be useful in industry and biotechnology.

Lipolytic enzymes are ubiquitous in nature, and microbial lipolytic enzymes are commercially significant [4,5]. In a classification scheme based on substrate preference, lipolytic enzymes are divided into lipases (EC 3.1.1.3) that hydrolyze long-chain acylglycerols ≥ 10 carbon chain), and esterases (EC 3.1.1.1) that hydrolyze short-chain acylglycerols ≤ 10 carbon chain). Both groups of biocatalysts have characteristics making them useful in a wide variety of industrial, pharmaceutical, biochemical, and biotechnological applications; *e.g.*, they have high chemo-, region- and stereo-selectivity, stability in organic solvents, usually do not require cofactors, and do not catalyze side reactions [6,7].

Lipolytic enzymes are serine hydrolases that share structural and functional characteristics such as an α/β hydrolase fold. Their catalytic mechanism involves a catalytic triad, or cofactor-independent activity [6]. Based on comparisons of amino acid sequences and biological properties, prokaryote-derived lipolytic enzymes have been classified into eight families, termed true lipases (family I), the enzymes display a Gly-Asp-Ser-(Leu) [GDS(L)] motif containing the active-site Ser (GDSL, family II), family III, hormone-sensitive lipases (HSL, family IV), and families V~VIII [4].

A culture-independent approach, termed "metagenomics" [8,9], allows screening for novel lipolytic enzymes, with industrial potential, from diverse environments [10]. For example, genes encoding lipolytic enzymes have been isolated from metagenomic libraries constructed from environmental samples including forest soils [11,12]; pond, lake, and river water [13-15] and hot spring and marine sediments [16,17]. With only a few exceptions, characteristics of the novel enzymes found so far are not very appropriate for industrial applications. Thus, further metagenomics-based search for novel lipolytic enzymes from different sources, and with greater industrial applicability, is an important task.

The offshore marine environment of the northern South China Sea, near the southern China continental shelf and Hainan Island (Additional file 1, Table S1), contains nutrient-rich waters with concentrations of organic compounds and diversity of marine microbes greater than those of most other regions of the open ocean. We collected sediment samples from this area, and performed functional screening for novel lipolytic enzymes using a metagenomic library.

## Results and discussion

### High efficient screening for lipolytic enzymes

Marine sediment samples from the South China Sea were collected from 23 sampling sites, depth < 100 m (Additional file 1, Table S1). A metagenomic library was constructed using ~2.1 μg of sediment DNA, and contained ~118,000 > 90%) recombinant colonies. Using 1217 recombinant plasmids, the library DNA insert size was estimated as 1.0 ~ 8.5 kb. The metagenomic library represented ~194 Mb of microbial community DNA of the marine sediment. A portion of the unamplified library (~60,000 colonies) was screened from screening plates. After 72 hr incubation at 37°C, 15 colonies were selected on the basis of stable hydrolysis zone and the lipolytic-positive plasmids were sequenced (Additional file 1, Table S2). The putative esterases, β-lactamases, phospholipases and patatin-like esterase were isolated from 49 identified genes.

It looks that pUC18 was useful vector for constructing small-insert metagenomic libraries, because of its high cloning and throughput screening efficiencies toward small-size target genes [18]. The pUC shotgun metagenomic library displayed the ability to rapidly assess large numbers of clones, avoiding the need for another sub-cloning library to obtain functional target genes.

### A novel lipolytic enzyme Est_p1

One clone showed strong lipolytic activity, and was designated as pNLE1 (EU628679). pNLE1 had an insert of 3650 bp, with 53.76% G+C content and four ORFs. Based on complete domain of α/β hydrolase fold-1 (PF00561) related to esterase/lipase superfamily, the ORF that encoded a 296 amino acids protein was identified as a putative lipase/esterase gene (designated as *est_p1*).

This encoded protein showed 81% amino acids identity with a lipolytic enzyme (ACF67850) from uncultured bacteria [19]. Most of the other close matches also came from uncultured bacteria of environmental samples obtained from deep-sea sediment in the South China Sea [19], or from soil of the Gwangneung forest in Korea [20]. None of these lipolytic enzymes were previously characterized. The closest match, aside from environmental samples, was an α/β hydrolase fold protein (YP_001310323) from *Clostridium beijerinckii *NCIMB 8052, also unpublished, showing 53% amino acids identity.

A putative ribosome binding site (RBS), GAGG, was detected upstream of the start codon at -12 to -9 region [21]. A strong promoter signal was found in the 2872-2827 bp range of pNLE1, located at the up-stream sequences of *nle1_3*, which might share the same promoter with Est_p1. SignalP3.0 analysis indicated that neither the cleavage site nor the N-terminal signal peptide was present in the whole gene [22], suggesting that Est_p1 may be expressed as a full-length protein, without requirement of flanking sequences or genes [23].

### Phylogenetic relationships of Est_p1

**Figure 1:**
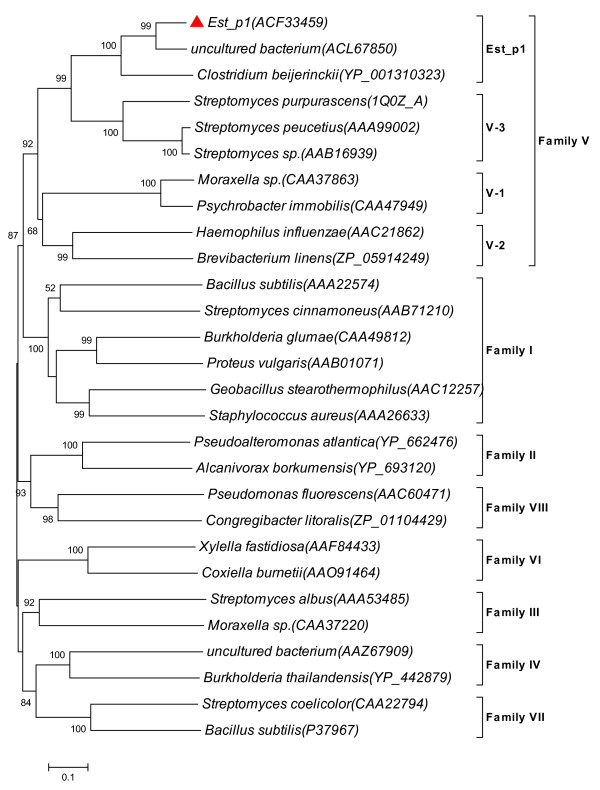
**Unrooted neighbor-joining phylogenetic tree of Est_p1 (red triangle) and relatives, based on conserved sequence motifs of bacterial lipolytic enzymes**. Amino acid sequences of other lipolytic enzymes were obtained from published data. Sequence alignment was performed using ClustalW version 2.0, and the tree was created by MEGA version 4.0. Scale bar at bottom indicates number of amino acid substitutions per site.

**Figure 2:**
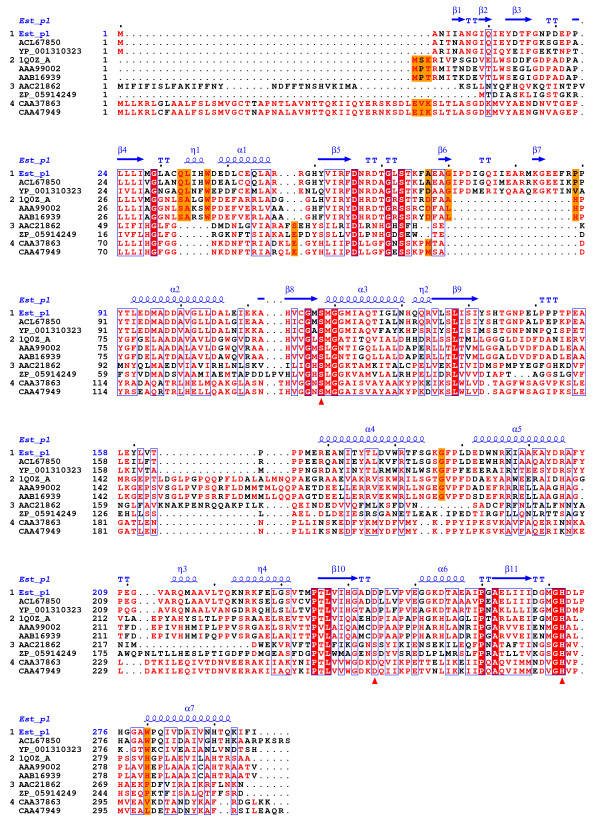
**Conserved sequence blocks from multiple sequence alignment between Est_p1 and family V members of known 3D structure**. Sequence alignment was performed using ClustalW version 2.0 and ESPript programs. Conserved sequences are indicated by box, and similar sequences are indicated by colored background. The catalytic triads are identical (red triangle). The alpha helix, beta sheet, random coil and beta turn are identical to α, β, η and T, respectively.

Multiple sequence alignment of Est_p1 and lipolytic proteins revealed the typical catalytic triad of active site serine (S^118^) motif G-x-S-x-G, conserved aspartic acid (D^244^), and histidine (H^272^) residue motif in the encoded protein. Bacterial lipolytic enzymes have been classified into distinct families on the basis of their amino acid sequences and biochemical properties [4]. In order to classify Est_p1, a phylogenetic tree was constructed using many lipolytic enzymes [24] representing eight different families. The results suggest that Est_p1 belongs to family V (Figure 1). Multiple sequence alignment of Est_p1 and family V members, including enzymes from cold-adapted organisms (*Moraxella *sp., *Psychrobacter immobilis*, 24-31% identity) [25,26], mesophilic bacteria (*Haemophilus influenza*, *Brevibacterium linens*, 23-24% identity) [27], methylesterase-producing bacteria (*Streptomyces purpurascens*, 33% identity) [28], solvent-producing bacteria (*Clostridium beijerinckii*, 53% identity) [29] and other uncultured bacteria (81% sequence identity) [19] showed relationship mainly to four subfamilies of family V. The alignment results showed three typical conserved motifs in Est_p1 and its subfamily. Of these, the η1 helix motif (Q-L-x-x-W, amino acid 33-37) and β6-7 stand motif (amino acid 67-89) may construct a characteristic cap structure in the subfamily (Figure 2). Members of the Est_p1 subfamily, which come from organic degradation organisms as above, are more likely to be related to each other than to other members of family V, and also show a functional relationship, *i.e.*, they specifically hydrolyze short-chain acylglycerols. Taken together, these findings indicate that Est_p1 is a new member of family V, belonging to a relatively independent subfamily.

### 3D model of Est_p1

**Figure 3:**
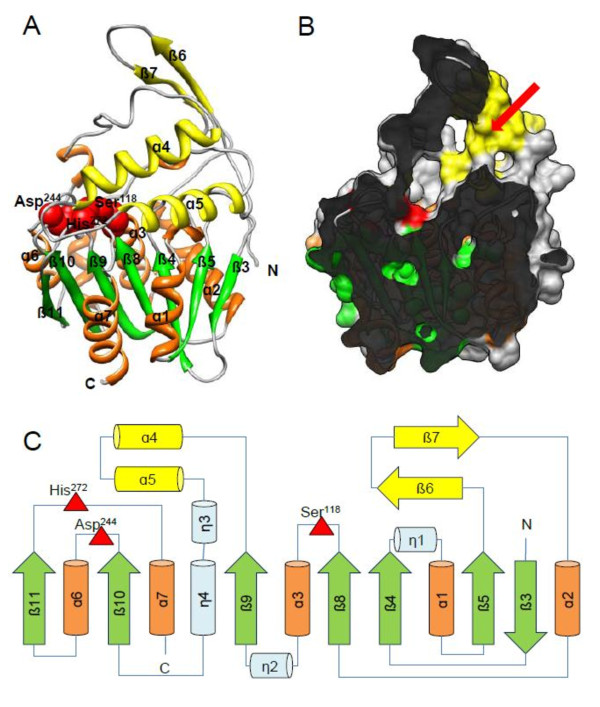
**3D model of Est_p1**. (A) Ribbon diagram of Est_p1 model. The central β-sheets and α-helices in core domains are shown in green and orange, respectively. The cap structure is shown in yellow. Residues of the catalytic triad (Ser^114^, Asp^244 ^and His^272^) are shown in red. (B) Cavity in the cap structure (shown in red arrow), acting as substrate binding pocket in Est_p1. (C) Topology diagram of Est_p1.

A model of Est_p1 was built using SWISS-MODEL Severs. Est_p1 displayed highest sequence homology with aclacinomycin methylesterase (accession code 1q0z) [28], with a modeled residue range from 4 to 293 amino acid (Figure 3A), and identity of 32%. Structurally, Est_p1 consisted of two domains. The core domain, which includes the catalytic triad (residues 4-58, 93-147, 222-293), contained 5 α-helices (α1-3, 6,7) and 7 β-sheets (β3-5, 8-11). The second domain consisted of 2 α-helices (α4, 5; residues 148-221) and 2 β-sheets (β6, 7; residues 59-92), which formed a cap structure over the α/β catalytic sheet. A substrate binding pocket, 17.7 Å long and 12.5 Å wide, was formed inside the cap structure (Figure 3B).

The catalytic triad, Ser^118^, Asp^244 ^and His^272^, were clustered close together at the bottom of this pocket. Ser^118 ^was located within a nucleophile "elbow" connecting sheet β8 and helix α3, while Asp^244 ^and His^272 ^were located on loops between β10-α6 and β11-α7, respectively (Figure 3C).

### Characterization of Est_p1

**Figure 4:**
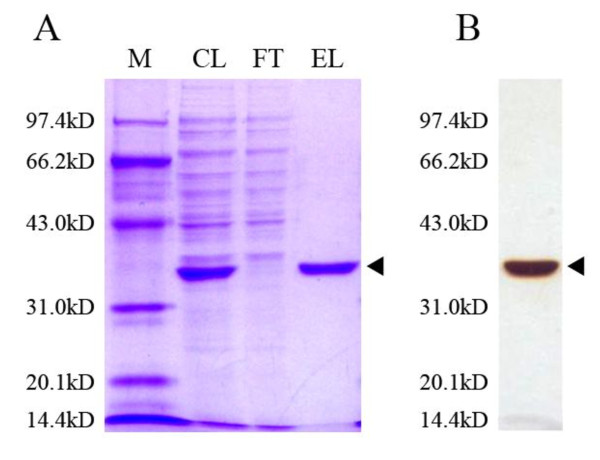
**Purification of recombinant Est_p1**. (A) Proteins recovered during various purification steps as described in the text were separated by SDS-10% polyacrylamide gel electrophoresis, and stained with Coomassie Brilliant Blue R-250. Lane M, molecular weight standards; Lane CL, induced cell lysate; Lane FT, flow-through; Lane EL, 50 mM imidazole elution. (B) Silver staining of purified Est_p1. Protein size markers are indicated (kilo Daltons, kDa) at left. Recombinant Est_p1 proteins are indicated by arrow at right.

The full-length *est_p1 *gene was amplified and cloned into pET28a with a C-terminal 6× His tag, then purified by Ni-NTA-agarose chromatography. The target protein appeared as a single band on SDS-PAGE with molecular weight ~35.5 kDa (Figure 4).

#### Optimal pH and pH stability

**Figure 5:**
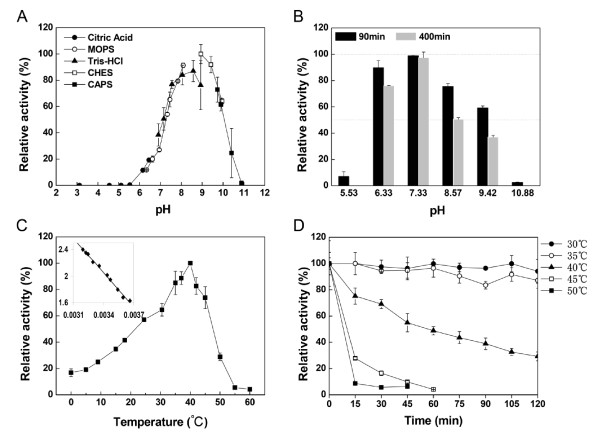
**Biochemical characterization of Est_p1**. (A) Effect of pH on Est_p1 activity. Est_p1 activity was measured at 40°C for 3 min in 50 mM buffer, at various pH levels. Values are shown as percentage of maximal activity, defined as 100%. Buffers used were sodium citrate (●), MOPS (○), Tris-HCl (▲), CHES (□), and CAPS (■). (B) Effect of pH on stability of Est_p1. Est_p1 was treated at 0°C for 90 min and 400 min in various buffers at various pH levels. Residual enzyme activity was measured at 40°C in 50 mM Tris-HCl buffer, pH 8.57. Values are shown as percentage of original activity (measured in the same buffer and pH, but without incubation at 0°C), defined as 100%. (C) Effect of temperature on Est_p1 activity. Activity was measured at various temperatures as indicated, for 3 min in 50 mM Tris-HCl buffer, pH 8.57. Values are shown as percentage of maximal activity, defined as 100%. The inset shows the temperature dependence as an Arrhenius plot. (D) Effect of temperature on stability of Est_p1. The enzyme was incubated in 50 mM Tris-HCl buffer, pH 8.57, at various temperatures, for 120 min, and residual activity was measured at 40°C for 3 min at 15 min intervals. Residual activity was expressed in Panel B.

Optimal pH and pH stability of purified Est_p1 were determined using C_4 _as substrate. Est_p1 displayed highest activity at pH values between 8 and 10, and optimal pH is 8.57 (Figure 5A). The apparent p*K*_a _was 7.5. The pH-dependent activity in serine carboxyl ester hydrolases is generally assumed to indicate involvement of the His residue in the catalytic triad [30]. However, no clear explanation for such mechanism has been established.

The pH stability was tested after incubation of purified Est_p1 for 90 to 400 min in various buffers at pHs between 3 and 11. After 90 min incubation, Est_p1 displayed > 70% residual activity in the pH range 6 to 8. At pH 6.33 and 8.57, after 400 min incubation, residual activity decreased by 13% and 25%, respectively, compared to that at 90 min (Figure 5B). At pH 7.33, after 400 min incubation, residual activity decreased by only 1% compared to that at 90 min. pH stability was therefore concluded to be greatest at pH 7.33.

#### Optimal temperature and thermostability

This determination was made using C_4 _as a substrate, at pH 8.57, with temperatures ranging from 0 to 60°C. Esterase activity increased as temperature increased up to 40°C, then decreased beyond that level. At temperatures above 55°C, there was essentially no enzyme activity. The optimal temperature was 40°C (Figure 5C).

From the linear part of the Arrhenius plot between 0 and 40°C, the activation energy for the formation of the enzyme/substrate complex was found to be 32.63 kJ/mol, similar to that of Pye3, another esterase of metagenomic origin [31]. The optimal temperature of Est_p1 was in the mesophilic range (40°C), like those of most esterases isolated from marine origins so far [32,33]. The optimal temperature for activity of an enzyme is usually higher than the optimal temperature for growth of the organism [19,34].

Thermostability of Est_p1 was determined by analysis of residual activity at regular intervals following pre-incubation of purified enzyme for durations up to 2 hr, at temperatures ranging from 30-50°C. Est_p1 was stable, with residual activity ~80%, after incubation at 30 or 35°C for 2 hr. At 40°C, the half-life was ~63 min (Figure 5D). At 45 or 50°C, residual activity dropped rapidly within 15 min. These findings suggest that Est_p1 originated from a mesophilic microorganism [30].

#### Substrate specificity

Lipolytic enzymes are characterized by the ability to hydrolyze a wide range of fatty acid esters. The distinction between lipase and esterase depends on specificity of aliphatic chain length [35]. To determine the substrate specificity of Est_p1, we tested its effect on various ρ-Nitrophenyl esters having acyl chain lengths of C_2_, C_4_, C_8_, C_10_, C_12_, C_16_, and C_18_, under assay conditions of pH 8.57 and 40°C. Est_p1 displayed hydrolytic activity for esters with short to medium chain length (C_2 _to C_10_; maximal for C_4_), but no detectable activity for esters with long chain length (C_12_, C_16_, C_18_) (Table 1). Lipases are defined by preference for substrates with long acyl chains; therefore, these findings indicate that Est_p1 is an esterase (EC. 3.1.1.1).

**Table 1:** Kinetic parameters for various ρ-Nitrophenyl esters of Est_p1

Substrate (ρ-Nitrophenyl ester)	*K*_m _(mM)	Vmax (μmol·min^-1^·mg^-1^)	*k*_cat _(S^-1^)	*k*_cat_/*K*_m _(S^-1^mM^-1^)
Acetate (C_2_)	0.585	244	315	543
Butyrate (C_4_)	0.858	2,260	9,850	11,500
Caprylate (C_8_)	0.348	109	140	401
Caprate (C_10_)	0.170	31.6	138	809

The α/β hydrolase fold enzymes are characterized by a nucleophilic "elbow" with the conserved motif G-x-S-x-G [36]. An enzyme's substrate specificity is determined by a flexible sequence that changes conformation with the binding pocket, defined by hydrophobic amino acid residues that line the pocket [28]. In the present study, the binding pocket surface of Est_p1 was not constructed by a large number of hydrophobic residues, suggesting that the pocket can accommodate only a limited number of carbon atoms [23].

Both the *K*_m _and *k*_cat _values of purified Est_p1 decreased as the acyl chain length increased up to C_4_. *K*_m _value indicates the affinity of substrate for enzyme. C_10 _had the lowest *K*_m _value (0.17 mM), suggested that C_10 _structure is closer to the natural substrate of Est_p1. However, Est_p1 showed a specific preference for C_4_, which had the highest *k*_cat_/*K*_m _value (11,500 S^-1^mM^-1^) of any of the substrates; this value was 21-, 28-, and 14-fold higher than that of C_2_, C_8_, and C_10_, respectively. C_4 _is the most appropriate substrate for Est_p1.

In general, there is a negative correlation between *K*_m _and *k*_cat _values for a particular enzyme toward different substrates, *i.e.*, a low *K*_m _value for a substrate indicates positive affinity for the enzyme, associated with higher catalytic activity and consequently a higher *k*_cat _value. In contrast, *K*_m _and *k*_cat _values for Est_p1 showed a positive correlation, and were both higher for the appropriate substrate, C_4_, than for other substrates tested. Similar results were found for other metagenomic esterases with heterologous expression, most of which were expressed by His tagging on the C- or N-terminus [31]. Addition of six hydrophilic His residues caused substrate specificity of the recombinant enzymes to shift toward more hydrophilic substrates [37].

The kinetic studies indicated that recombinant Est_p1 had high specificity (*k*_cat_/*K*_m _value 11,500 S^-1^mM^-1^) for substrate C_4_. This value is much higher than those reported for other metagenomic esterases, *e.g.*, those from sludge samples at a South African mine refinery (7.65 S^-1^mM^-1^) [23], Antarctic desert soil (14.8 S^-1^mM^-1^) [38], tidal flat sediments (18.2 S^-1^mM^-1^) [39], marine environment (26.7 S^-1^mM^-1^) [16], hot springs in Tangkuban Perahu (2,290 S^-1^mM^-1^) [40] and hot springs in Thailand (4,101.2 S^-1^mM^-1^) [17].

Esterase is commonly used in production of enhancement the buttery flavor of the end product. Moreover, these enzymes which specificity towards short chain fatty acids, especially butanoic acid, are commercially available as potent flavoring tools [41]. In view of its high specificity and hydrolysis activity, Est_p1 has potential application in flavor industries requiring hydrolysis or synthesis of short chain esters.

#### Effects of solvents, detergents, metal ions and EDTA on Est_p1 activity

The activity of Est_p1 in various solvents and detergents was examined, as summarized in Table 2. In the case of solvents and detergents, SDS and PVPP inhibited enzymatic activity of Est_p1 at the concentration of 2 mM, whereas others displayed no significant effect on the hydrolytic activity of Est_p1.

**Table 2:** Effect of organic solvents and cations on activity of Est_p1

Solvent	Relative activity (%)			Cations	Relative activity (%)		
			
	0.5 mM	1 mM	2 mM		0.5 mM	1 mM	2 mM
GITC	113 ± 0.8	104 ± 1	93.7 ± 8	Mg^2+^	97.6 ± 12	101 ± 0.03	86.7 ± 3
PMSF	104 ± 2	110 ± 3	99.5 ± 8	Ca^2+^	96.3 ± 3	86.5 ± 1	55.1 ± 3
PVPP	85.6 ± 3	73.5 ± 3	58.6 ± 3	Mn^2+^	78.0 ± 6	85.5 ± 3	63.5 ± 2
SDS	99.5 ± 2	71.5 ± 0.6	30.0 ± 1	Co^2+^	82.3 ± 6	79.6 ± 14	12.0 ± 2
Thiourea	105 ± 1	95.6 ± 6	95.3 ± 0	Ni^2+^	54.4 ± 5	39.1 ± 7	24.4 ± 4
Urea	102 ± 0.6	105 ± 2	101 ± 2	Zn^2+^	24.1 ± 10	24.6 ± 5	24.4 ± 11
EDTA	110 ± 18	113 ± 6	108 ± 5	Cu^2+^	19.1 ± 0.1	11.3 ± 2	4.11 ± 1

In the case of metal ions, the higher concentrations displayed progressively greater inhibitory effect on Est_p1 activity, roughly in proportion to molecular weight. On the other hand, EDTA had no significant influence on Est_p1. The finding indicates that Est_p1 does not require the presence of co-factors, which also confirmed the Est_p1 has no metal-binding site structurally.

Lipolytic enzymes belong to the class of serine hydrolases, and their activity is generally found to be irreversibly inhibited by PMSF. However in our study, PMSF had no effect on catalytic activity of Est_p1, which was also rarely found in other two lipolytic enzymes [24,42]. Our finding suggests that the inhibitory effect of PMSF was eliminated by a lid structure in carboxylesterases [43]. The 3D model of Est_p1 (Figure 3) predicts that the entrance to the binding site is sheltered by β6 and β7, which act as a lid structure to protect the catalytic serine residue. Aromatic residues surrounding the serine (*e.g.*, His, Trp) may play a steric role to prevent modification by PMSF.

## Conclusions

Based on marine sediment metagenomic library was successfully constructed, a novel family V esterase gene, termed *est_p1 *was cloned and expressed in *E. coli*. The recombinant Est_p1 efficiently catalyzed hydrolysis of substrates with short-chain esters at pH 8.57, 40°C, and acts as an esterase (EC. 3.1.1.1). It was not sensitive to PMSF and does not require metal co-factors. The discovery that Est_p1 has high specificity and hydrolysis activity towards ρ-Nitrophenyl butyrate (C_4_), whose value was 21-, 28-, and 14-fold higher than that of C_2_, C_8_, and C_10_, demonstrating Est_p1 has potential application in flavor industry requiring hydrolysis or synthesis of short chain esters. Further study on application of the enzyme for releasing short chain fatty acids from the low flavor fat-rich matrices in order to enhance its highly appreciated flavor will be further investigated. In addition, further studies may provide important data for future application of the novel metagenomic esterases for promising biotechnological processes.

## Materials and methods

### Strains, plasmids, and marine sediment samples

*Escherichia coli *DH5α and vector pUC18 were used for library cloning, *E. coli *BL21 (DE3) and pET-28a (+) (Novagen) was used for heterologous expression of target protein.

Marine sediment samples from the South China Sea were collected from 23 sampling sites, depth < 100 m (Additional file 1, Table S1) and stored at 4°C until DNA extraction.

### Metagenomic library construction and functional screening of lipolytic clones

Metagenomic DNA was isolated according to described previously [44]. After purified DNA by pulsed field gel electrophoresis (PFGE) (Bio-Rad CHEP Mapper XA, fixed angle 120, 4.5 V/cm, running time 15 hr, 1-12 sec switch, 15°C), and the DNA was partially digested with *Sau*3AI. The recovered DNA fragments (size 2-9 kb) were ligated into pUC18 vectors, and then electroporated in *E. coli *DH5α. Lipolytic clones were detected based on their ability to hydrolyze tributyrin (1%) substrate, and to produce a clear halo around the colony after 48 hr incubation at 37°C. All lipolytic clones were streaked to obtain single colonies, and re-tested for ability to hydrolyze tributyrin [14].

### Bioinformatic analysis

The positive clones were confirmed by plasmid isolation and restriction enzyme digestion, and the unique plasmids were sent to a DNA sequencing facility (Invitrogen, Beijing, China) for primer-walking sequencing approach.

Sequences were screened for vector contamination and quality trimmed. Assembly and analysis were performed using DNAMAN (version 6.0, Lynnon Corp., Canada) and GENETYX (version 8.01, Genetyx Corp., Japan) programs, respectively. Open reading frames (ORFs) in each assembled sequence were identified using the ORF Finder at the National Centre for Biotechnology Information (NCBI) website. Amino acid sequences of each ORF were used to find the best match, and conserved domains, by protein-protein BLAST program at the NCBI website. The subfamily and superfamily of each encoded protein was determined by searching the Lipase Engineering Database [45,46]. Signal peptide and transmembrane domain were predicted using server SignalP and HMMTOP. Promoter prediction was conducted using Neural Network Promoter Prediction (NNPP) version 2.2. Positional frequency matrices (PFMs) of *E. coli *promoters were also used to predict positions of possible promoters [47]. Ribosome binding site (rbs) prediction was conducted using the PFMs [48]. Multiple sequence alignments were calculated using ClustalW and exported by ESPript. Phylogenetic relationships among lipolytic members in each protein family were analyzed by MEGA 4.0 [4]. The 3D model of Est_p1 was constructed by SWISS-MODEL http://swissmodel.expasy.org/ and presented using UCSF Chimera, version 1.4.1. The size of banding pocket was measured by the distances of atoms located at the pocket edge and also calculated by UCSF Chimera, version 1.4.1.

### Expression and purification of Est_p1

Full-length *est_p1 *gene was amplified from the plasmid nle1. The forward primer (5'-CATG**CCATGG**CAAACATTATTGCG-3') with the restriction enzyme site *Nco*I, and the reverse primer (5'-ACGC**GTCGAC**GATA AAAATTTTTTGGGT-3') with *Sal*I, were designed to generate a C-terminal His-tag of the recombinant target protein. The *est_p1 *gene was cloned into expression vector pET-28a (+), and was transformed into *E. coli *BL21 (DE3) cells. Transformants were grown on LB medium containing 50 μg ml^-1 ^kanamycin at 37°C. When cells reached a density in the 0.5-1.0 range at 600 nm, they were induced for 16 hr with 0.5 mM IPTG at 30°C.

The target protein was purified by Ni-NTA (Qiagen) affinity chromatography, and protein concentration was determined using Lowry protein assay method, with BSA protein as standard. Purity of the protein was confirmed by SDS-PAGE, and protein bands were visualized by Coomassie Brilliant Blue R-250 and silver staining.

### Enzyme characterization

Lipase/esterase activity was determined by a spectrophotometric method using ρ-Nitrophenyl (ρNP) esters. Catalytic activity of Est_p1 was examined using ρNP butyrate as standard substrate (unless indicated otherwise) at 40°C for 3 min. The assay mixture contained 1 mM ρNP esters, 50 mM Tri-HCl buffer (pH 8.57), and 4% ethanol, in a total volume of 1 ml. Absorbance was measured at 405 nm. One unit esterase was defined as the amount of enzyme needed to liberate 1 μmol ρNP in 1 min.

Optimal pH of purified Est_p1 was determined under standard conditions. Buffers used were 50 mM of sodium citrate (pH 3.12 - 6.45), MOPS (pH 6.33 - 8.09), Tris-HCl (pH 6.9 - 8.93), CHES (pH 8.94 - 9.95) and CAPS (pH 9.73 - 10.88). The pH stability was determined by incubating the assays at various pH (5.53 to 10.88) for 90 min and 400 min, and the residual activity was measured.

Optimal temperature was measured under standard conditions, in the range 0 - 60°C. Thermostability was determined by incubating the assays at temperatures ranging from 30 to 50°C for 120 min, and measuring residual activity.

Substrate range and specific activity were determined under standard conditions using ρNP esters with acyl-chains of various lengths: ρNP acetate (C_2_), ρNP butyrate (C_4_), ρNP caprylate (C_8_), ρNP caprate (C_10_), ρNP laurate (C_12_), ρNP palmitate (C_16_), ρNP stearate (C_18_). Initial reaction velocities measured at various substrate concentrations were fitted to the Lineweaver-Burk transformation of the Michaelis-Menten equation. Kinetic analyses by curve fitting were performed with the Fit linear program (OriginLab Corp., USA).

Activity of purified Est_p1 was assayed under standard conditions in the presence of various potentially inhibitory reagents: divalent metal cation (Mg^2+^, Ca^2+^, Mn^2+^, Co^2+^, Ni^2+^, Cu^2+^, Zn^2+^) (0.5, 1 and 2 mM), chelating agent (EDTA), inhibitor (PMSF), detergents (SDS, guanidine thiocyanate, thiourea, urea), and polar affinitive surfactant polyvinylpoly-pyrrolidone (PVPP) (0.5, 1 and 2% w/v).

### Nucleotide sequence accession number

The amino acid sequence of Est_p1 is available at the GenBank database [GenBank: ACF33459].

## Abbreviations

GDSL: motif consensus amino acid sequence of Gly, Asp, Ser, and Leu around the active site Ser; PMSF: phenylmethylsulfonyl fluoride; GITC: guanidine thiocyanate; PVPP: polyvinylpolypyrrolidone; SDS: sodium dodecyl sulfate; EDTA: ethylenediaminetetraacetic acid; C_2_: ρ-Nitrophenyl acetate; C_4_: ρ-Nitrophenyl butyrate; C_8_: ρ-Nitrophenyl caprylate; C_10_: ρ-Nitrophenyl caprate; C_12_: ρ-Nitrophenyl laurate; C_16_: ρ-Nitrophenyl palmitate; C_18_: ρ-Nitrophenyl stearate.

## Competing interests

The authors declare that they have no competing interests.

## Authors' contributions

YL, GHG, QP initiated and coordinated the project. QP and XZH performed construction of metagenomic library. QP, MS and XW performed gene cloning and expression in *E. coli*. QP, GLW and GHG were responsible for enzyme characterization. YL, BXL, GHG and YSW provided critical discussion. QP and YL wrote the paper and all authors approved the final version of the manuscript.

## Supplementary Material

Additional file 1 **Table S1 Designation, coordinates and depth of 23 marine sediment samples collected in the South China Sea**. The information of marine sediment sample source. **Table S2 Lipolytic enzymes from metagenomic library and compared to homologous proteins in GenBank**. 15 Lipolytic enzyme genes were cloned from metagenomic library and their accession numbers in GenBank. Click here for file
