# Supplementary material for: A novel esterase gene cloned from a metagenomic library from neritic sediments of the South China Sea
Source: Microb Cell Fact. 2011 Nov 9;10:95. doi: 10.1186/1475-2859-10-95 (PMC3226443; doi:10.1186/1475-2859-10-95)
Supplement: Additional file 1 — Table S1 Designation, coordinates and depth of 23 marine sediment samples collected in the South China Sea. The information of marine sediment sample source. Table S2 Lipolytic enzymes from metagenomic library and compared to homologous proteins in GenBank. 15 Lipolytic enzyme genes were cloned from metagenomic library and their accession numbers in GenBank. [file 1475-2859-10-95-S1.DOC]

**Additional file1**

**Table** S**1** Designation, coordinates and depth of 23 marine sediment samples collected in the South China Sea

| Sample | Latitude (N) | Longitude (E) | Depth (m) |
| --- | --- | --- | --- |
| D13-2 | 21˚21.051' | 111˚31.484' | 28 |
| D13-4 | 21˚0.391' | 111˚47.041' | 45 |
| D13-6 | 20˚38.260' | 112˚3.361' | 69 |
| D13-8 | 20˚13.038' | 112˚24.517' | 89 |
| D14-2 | 21˚10.124' | 110˚57.823' | 20 |
| D15-2 | 20˚50.652' | 110˚44.419' | 29 |
| D15-4 | 20˚34.529' | 110˚56.821' | 35 |
| D15-5 | 20˚24.890' | 111˚4.820' | 46 |
| D15-8 | 20˚0.091' | 111˚25.058' | 75 |
| D16-1 | 20˚10.345' | 111˚02.976' | 38 |
| D16-2 | 20˚01.509' | 111˚10.014' | 52 |
| D16-3 | 19˚50.603' | 111˚18.909' | 75 |
| D17-3 | 19˚25.610' | 111˚11.999' | 85 |
| D18-3 | 19˚04.328' | 110˚58.445' | 96 |
| D19-1 | 18˚49.876' | 110˚36.366' | 89 |
| D20-1 | 18˚36.089' | 110˚17.537' | 29 |
| D20-2 | 18˚33.077' | 110˚27.886' | 55 |
| D21-1 | 18˚19.940' | 109˚52.210' | 53 |
| D21-2 | 18˚17.730' | 110˚8.105' | 85 |
| D21-3 | 18˚10.417' | 110˚14.648' | 99 |
| D22-1 | 18˚8.226' | 109˚42.427' | 78 |
| D22-3 | 17˚57.113' | 109˚49.903' | 89 |
| D22a-2 | 17˚54.190' | 109˚37.605' | 82 |

**Table S2** Lipolytic enzymes from metagenomic library and compared to homologous proteins in GenBank

| Clone | | | | Homologous Protein | | |
| --- | --- | --- | --- | --- | --- | --- |
| Name | ORF GC (%) | ORF (aa) | AC. No.* | Best match (AC. No.) | Organism | Identity (%) |
| Est_p1 | 53.7 | 296 | ACF33459 | lipolytic enzyme (ACL67850) | Top of Form  uncultured bacteriumBottom of Form | 81 |
| Est_p2 | 56.5 | 252 | ACF33453 | acyl-CoA thioesterase I (YP_693120) | *Top of Form*  *Alcanivorax borkumensis* SK2 Bottom of Form | 65 |
| Est_p3 | 63.4 | 305 | ACF33456 | putative esterase (ZP_00956786) | *Top of Form*  *Sulfitobacter sp.* EE-36Bottom of Form | 97 |
| Est_p4 | 66.8 | 410 | ACZ16561 | beta-lactamase family protein (YP_002518666) | *Top of Form*  *Caulobacter crescentus* NA1000Bottom of Form | 59 |
| Est_p5 | 63 | 386 | ACZ16562 | beta-lactamase (ZP_00956527) | *Top of Form*  *Sulfitobacter sp.* EE-36Bottom of Form | 98 |
| Est_p6 | 59.5 | 357 | ACZ16565 | putative esterase (YP_002394831) | *Vibrio splendidus* LGP32 | 59 |
| Est_p7 | 68.6 | 299 | ACZ16567 | lipase/esterase (AAX37296) | Top of Form  uncultured bacteriumBottom of Form | 49 |
| Est_p8 | 57.2 | 404 | ACZ16570 | Beta-lactamase (ZP_01736890) | *Top of Form*  *Marinobacter sp.* ELB17Bottom of Form | 76 |
| Est_p9 | 61.8 | 254 | ACZ16573 | α/β fold family protein (ZP_00948319) | *Top of Form*  *Sulfitobacter sp.* NAS-14.1Bottom of Form | 97 |
| Est_p10 | 64 | 391 | ACZ16575 | patatin (YP_532891) | *Top of Form*  *Rhodopseudomonas palustris* BisB18Bottom of Form | 34 |
| Est_p11 | 46.7 | 226 | ACZ16579 | Carboxylesterase (YP_003145361) | *Kangiella koreensis* DSM 16069 | 78 |
| Est_p12 | 53.6 | 378 | ADQ39292 | beta-lactamase (ZP_05095337) | Top of Form  marine gamma proteobacterium HTCC2148Bottom of Form | 69 |
| Est_p13 | 63.2 | 304 | ADQ39294 | putative esterase (ZP_00956786) | *Top of Form*  *Sulfitobacter sp.* EE-36Bottom of Form | 97 |
| Est_p14 | 64.4 | 202 | ADQ39298 | acetyltransferase (YP_002354215) | *Top of Form*  *Thauera sp.* MZ1TBottom of Form | 43 |
| Est_p15 | 38.8 | 229 | ADQ39285 | hypothetical protein GFO_1069 (YP_861110) | *Top of Form*  *Gramella forsetii* KT0803Bottom of Form | 58 |

*AC. No.: Accession number in GenBank
